# Small extracellular vesicles from follicular fluid as transport carriers of *LHR*: possible mediators of follicle growth and dominance acquisition in bovine reproduction

**DOI:** 10.1590/1984-3143-AR2025-0051

**Published:** 2026-01-09

**Authors:** Luca Angi Souza, Nico G. Menjivar, Ahmed Gad, Paulo Henrique Groppo Rodrigues, Letícia Rabello da Silva Sousa, Paola Maria da Silva Rosa, Alessandra Bridi, Dawit Tesfaye, Juliano Coelho da Silveira

**Affiliations:** 1 Departamento de Medicina Veterinária, Faculdade de Zootecnia e Engenharia de Alimentos, Universidade de São Paulo – USP, Pirassununga, SP, Brasil; 2 Animal Reproduction and Biotechnology Laboratory – ARBL, Department of Biomedical Sciences, College of Veterinary Medicine and Biomedical Sciences, Colorado State University, Fort Collins, USA; 3 Department of Animal Production, Faculty of Agriculture, Cairo University, Giza, Egypt; 4 Universidade do Oeste de Santa Catarina, Xanxerê, SC, Brasil

**Keywords:** luteinizing hormone receptor, mRNA, extracellular vesicles, cargo transfer, follicular development

## Abstract

Luteinizing hormone (LH) plays a crucial role in follicle development, ovulation induction, and the regulation of key reproductive events. However, the efficacy of LH within the follicular microenvironment largely depends on the capacity of follicular cells to express its receptor. This study aims to investigate whether granulosa cells (GCs) can acquire *LHR* through extracellular vesicles (sEVs) present in follicular fluid (FF) from follicles of varying sizes. In the first experiment, GCs and sEVs were collected from the FF of small (3–5 mm), medium (5.1–7 mm), and large (7.1–9 mm) ovarian follicles from *Bos taurus indicus* cows. In the second experiment, GCs and sEVs were collected from the FF of small (3–6 mm) and large (8–14 mm) follicles from *Bos taurus taurus* cows. Initially, we assessed the ability of sEVs to carry *LHR* mRNA by comparing its expression profiles in sEVs derived from different size follicles. Our findings revealed that as follicular development progresses, *LHR* levels in FF sEVs decrease, while in corresponding GCs, from which the sEVs primarily originate, show increased *LHR* expression. To further investigate whether GCs represent an additional source of FF sEVs carrying LHR mRNA, GC cultures were established and sEVs secreted into the culture medium (ME-sEVs) were analyzed for *LHR* mRNA levels. A similar pattern was observed in ME-sEVs derived from GCs of small versus large follicles, with decreased LHR mRNA levels in sEVs secreted by GCs from large follicles compared to small follicles. This suggests that *LHR* is likely packaged into sEVs in small follicles stage, and shuttled into follicular cells during follicular growth, preparing them for the ovulatory stimulus. Our study uncovers a possible mechanism of *LHR* acquisition by GCs, which involves EVs and can possibly be involved in follicle quality and ability to respond to LH stimulus.

## Introduction

In dairy cattle, ovarian dysfunction significantly impairs reproductive efficiency, affecting approximately 10-50% of cows across herds. This condition leads to delayed ovulation and reduced conception rates (Ferguson, 2017). During folliculogenesis, the oocyte undergoes several modifications facilitating its release from the ovary and transition into the oviduct, where fertilization by the male gamete can occur. Hormonal regulation predominantly governs the bovine estrous cycle, influencing follicular development, including the establishment of follicular dominance or the regression of subordinate follicles (Noseir, 2003).

Luteinizing hormone (LH) is a key regulatory hormone whose levels fluctuate throughout the estrous cycle. One of LH's critical roles is to promote the continued growth of follicles beyond 9 mm and facilitate subsequent ovulation (Gong et al., 1996). However, the proper function of LH requires the presence of its receptors in follicular cells, such as GCs and theca cells. It has been previously reported that dominant follicles failing to ovulate are characterized by reduced estradiol production and a decreased number of LH receptors (Ferguson, 2017). Abnormalities in LH receptor presence and function underpin several reproductive anomalies in cattle, which may also have implications for humans, leading to genetic disorders manifesting as amenorrhea, oligomenorrhea (Latronico and Arnhold, 2012), and infertility (Arnhold et al., 1999). Despite these findings, the mechanisms underlying LH receptor development in GCs and the progression of ovarian follicles to attain dominance remain unclear. Therefore, elucidating the factors involved in LH receptor acquisition necessitates an in-depth understanding of the gene expression encoding the LHR. The LHR has been identified in the literature as a marker gene of interest for oocyte quality. It is known to be more abundantly expressed in GCs from follicles containing viable oocytes, potentially indicating follicular viability (Matoba et al., 2014). Abundant transcript levels of LHR have been notably observed in GCs of dominant follicles compared to subordinate ones (Beg et al., 2001; Evans et al., 2004). The presence of these transcripts, and consequently the abundance of LH receptors, varies in dominant follicles in response to FSH secretion and LH increases (Landry and Sirard, 2018). Thus, the induction of LHR expression in GCs is essential for follicular maturation, facilitating the progression of follicles towards dominance (Pakarainen, et al., 2007). Conversely, subordinate follicles within a given follicular wave tend to undergo regression or inhibited growth, eventually entering atresia, contingent on the status of the dominant follicle (Ginther et al., 2003). Understanding follicular growth and the acquisition of LHR in follicular cells during folliculogenesis is crucial to unraveling the mechanisms by which intercellular communication mediates LHR acquisition within the follicular microenvironment. Among the various intercellular communication strategies, extracellular vesicle-mediated cellular crosstalk enables the transfer of bioactive molecules from donor to recipient cells.

Extracellular vesicles are nano-sized, phospholipid-enveloped particles secreted by almost all cell types, and abundantly present in most biological fluids (Théry et al., 2009). Notably, one of the most promising and definitive characteristics of sEVs is their capacity to shuttle important regulatory bioactive materials, such as mRNAs, microRNAs, and proteins, which govern critical pathway functions from one cell to another (Simpson et al., 2008). In reproductive function, EV-mediated intercellular communication is known to be involved in processes such as oocyte maturation (Lange-Consiglio et al., 2017), fertilization (Al-Dossary et al., 2013), and early embryonic development (Lopera-Vasquez et al., 2016). Furthermore, we have previously demonstrated that FF sEVs are abundantly uptake by GCs both *in vivo* and *in vitro* (Silveira et al., 2018), underscoring their potential role in mediating cell-cell communication within the ovarian follicle.

Essentially, we know that LH plays a very critical role in oocyte maturation (Pandey et al., 2010) well as ovulation (Gong et al., 1996). The *LHR* gene, which encodes LH receptor, has it levels increased in GCs from follicles since 5 mm of diameter until higher than 10 mm; while in the theca cells it is maintained during the entire follicle development (Nogueira et al., 2007). The FF sEVs are known to be able to transmit messages among follicular cells, which could promote the oocyte development (Andrade et al., 2017). Additionally, the mechanism by which follicular fluid sEVs transfer mRNAs and/or proteins between follicular cells has already been proposed (Silveira et al., 2014). Taken together, we hypothesize that sEVs from small ovarian follicles have higher levels of *LHR*, while the opposite occurs in granulosa cells. This pattern suggests that LH receptor acquisition may not occur solely through endogenous expression but also through transcripts acquired via sEV uptake.

## Methods

**Figure 1:**
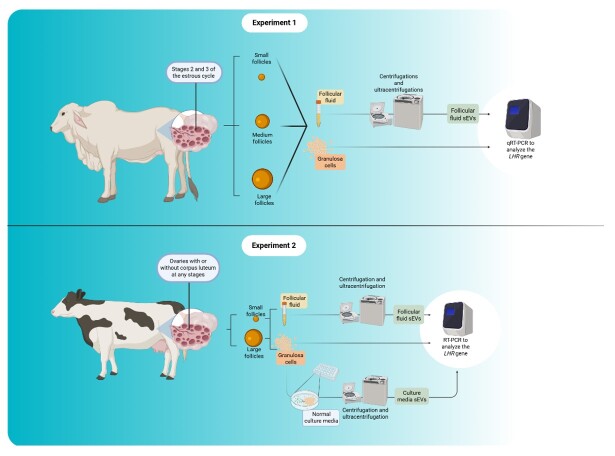
Schematic representation of the experimental design for the Experiments 1 and 2. In the first experiment, the FF sEVs and the GCs from small (3-5 mm), medium (5.1-7 mm) and large (7.1-9 mm) follicles from ovaries of the stage 2 and 3 of the estrous cycle (mostly from *Bos taurus indicus*) had their *LHR* transcripts evaluated. In the Experiment 2, the ovaries were only from *Bos taurus taurus* and it was collected with or without the corpus luteum at any stages of the estrous cycle. Firstly, small (3-6 mm) and large (8-14 mm) follicle sEVs and the GCs also had their *LHR* transcripts evaluated. Finally, the GCs from small and large follicles of random ovaries were cultured in normal conditions and had the sEVs from the culture media isolated and analyzed for *LHR* transcripts 48 hours after the cells were washed.

This study comprises two experiments conducted at different universities, utilizing ovaries from cows of distinct breeds (Figure 1). Experiment 1 was performed in Brazil at the Laboratory of Molecular Morphophysiology and Development (LMMD) at the University of São Paulo (Pirassununga – SP). The ovaries came from slaughterhouses and animals crossed with *Bos taurus indicus*. The experimental procedure using cattle was approved by the University of São Paulo Research Ethics Committee (protocol number: 4166120117) and complied with the ethical principles of animal research.

Experiment 2 was performed in the United States of America, at the Animal Reproduction and Biotechnologies Laboratory (ARBL) at the Colorado State University (Fort Collins – CO). The ovaries that came from the slaughterhouse were from *Bos taurus taurus*, different from the samples collected in Experiment 1. Thus, the LHR1 primer (used in Experiment 1) had to be changed to LHR2 (used in Experiment 2) (Table 1).

**Table 1:** Bovine primer data used in the qRT-PCR amplification for the Experiments 1 (*LHR1*, *YWHAZ* and *GAPDH1*) and 2 (*LHR2*, *GAPDH2* and *ß-ACT)*.

**Gene symbol**	**Primer sequences (5′-3′)**	**Product size**	**Accession no.**
*LHR1*	FP: TCTCAGAGTGATTCCCTGGAAAAGA	109	NM_174381.1
	RP: CAGCCTCAATGTGCACCAGG		
*YWHAZ*	FP: GCATCCCACAGACTATTTCC	120	GU817014.1
	RP: GCAAAGACAATGACAGACCA		
*GAPDH1*	FP: GCCATCAATGACCCCTTCAT	70	NM_001034034.2
	RP: TGCCGTGGGTGGAATCA		
*LHR2*	FP: AAACATGCCATTCCAGTCAT	280	NM_174381
	RP: GGACTGCCATTTTCTTAGCA		
*GAPDH2*	FP: AATGGAAAGGCCATCACCATC	204	NM_001034034.2
	RP: GTGGTTCACGCCCATCACA		
*ß-ACT*	FP: TGTCCACCTTCCAGCAGAT	249	NM_173979
	RP: TCACCTTCACCGTTCCAGT		

### Experiment 1

#### Collection of granulosa cells and follicular fluid

In the experiment 1, bovine ovaries were collected from slaughterhouses, placed immediately in saline solution at 37ºC and transported to the laboratory within 3 hours. In order to restrict analysis the ovaries collected in pairs at the slaughterhouse were classified in stages from 1 to 4 (Ireland et al., 1980), and only stages 2 and 3 were used for the experiment. This restriction was decided in order to avoid large variations due to different endocrine follicular environments. The ovarian follicles were individually dissected and measured using a caliper. Later, the follicles were separated in three groups based on their diameters (3–5 mm—small, 5.1–7 mm—medium and 7.1–9 mm—large follicles), dissected and ruptured individually under the stereoscope. After rupturing the follicles in petri dishes, the GCs (sheet like) and the FF were collected. The GCs were centrifuged twice at 500×g for 5 minutes and the pellet was collected. FF was collected for FF sEVs isolation. Briefly, the FF was centrifuged at 4 ºC in order to remove live cells (300×g for 10 min), cellular debris (2,000×g for 10 min), and large EVs (16,500×g for 30 min). Once processed, the FF and GC samples were snap frozen in liquid nitrogen and stored at -80ºC until further analysis.

#### Isolation of small extracellular vesicles from follicular fluid

After serial centrifugations, the FF was filtered through a 0.20 µm sterile syringe filter (PES membrane; Corning) to remove any remaining large EVs. The fluid included pooled samples from approximately 3 small follicles or from single medium or large follicles to reach a minimum volume for analysis. Each experimental group consisted of approximately 7 biological replicates, with all pools containing samples from different animals. The samples were then centrifuged twice at 119,700×g for 70 minutes (Optima XE-90 Ultracentrifuge; rotor 70 Ti; Beckman Coulter, Brea, California, USA) to isolate FF sEVs. Before ultracentrifugation, the volume was equalized by adding phosphate-bufered saline without Ca^2+^/Mg^2+^ (PBS−) to ensure consistency. The supernatant was discarded, and the FF sEVs pellet was resuspended in 20 µL of PBS− until further use.

#### Nanoparticle tracking analysis

The particle size and concentrations were measured using a Nanosight (NS300; NTA 3.1 Build 3.1.45, Malvern, UK). For the analysis, 5 µL of the 20 µL resuspended sEVs pellet was diluted in 995 µL of PBS−. The analysis involved capturing five 30-second videos for each sample, using an sCMOS camera set at level 15, with a threshold of 5. All measurements were conducted at a controlled temperature of 38.5 °C.

#### Transmission electron microscopy

Small extracellular vesicles pellets isolated from approximately 40 μl of FF were diluted in 200 μL of fixing solution (0.1 M cacodylate; 2.5% glutaraldehyde and 4% paraformaldehyde at pH 7.2–7.4) for 2 hours at room temperature. Subsequently, the FF sEVs were diluted in 2 mL of nuclease-free water, and the solution was ultracentrifuged once to obtain pellets of FF sEVs (119,700×g, 70 min, 4 ◦C). The pellet was diluted in 20 μL of nuclease-free water and placed in a copper grid for 20 minutes at room temperature for it to dry before staining. The grid was inserted into 2% of uranyl acetate and then analyzed using a transmission electron microscope (FEI Tecnai 20; LAB6 emission; 200 kV).

#### Nano-flow cytometry

Flow cytometry was employed to analyze extracellular vesicle (EV) markers. To optimize machine performance and minimize background noise, ultrapure water was used as the sheath fluid. The isolated EVs were labeled with the following antibodies as positive markers: PE-conjugated mouse monoclonal ALIX (sc-53540; 1:50), FITC-conjugated mouse monoclonal CD9 (AB18241; 1:10), and FITC-conjugated mouse monoclonal CD63 (AB18235; 1:10). To assess potential contamination in the isolates, Calnexin (sc-23954; 1:50) was included as a control marker. For sample preparation, pooled S-EVs and L-EVs were incubated with the antibodies for approximately 2 hours at room temperature under constant agitation. Prior to incubation with ALIX and Calnexin, the EV samples were treated with a 0.001% Triton X-100 (X100, Sigma-Aldrich) solution for 15 minutes at room temperature to enhance membrane permeability. Following antibody incubation, the samples were diluted in 200 µL of PBS, triple-filtered, and analyzed using a CytoFLEX flow cytometer (Beckman Coulter). Instrument settings were optimized for nanoparticle detection using the violet SSC channel (V-SSC) and PE fluorophore. The V-SSC gain was set to 100, FITC to 450, and PE to 600. The primary threshold was established at 500 for V-SSC, with a secondary threshold of 600 for FITC. Sample acquisition was conducted at a flow rate of 10 µL/min for 5 minutes per sample, maintaining an event rate of approximately 2000 events/s and an abortion rate below 8%. Nanoparticle size estimation was performed using a mixture of fluorescent Megamix-Plus SSC and Megamix-Plus FSC beads (BioCytex) with defined diameters of 100, 160, 200, 240, 300, 500, and 900 nm. Gating was adjusted based on negative control samples to ensure the exclusion of background signals. The number of detected events within the defined gates was used to determine marker expression in the EV samples.

#### Total RNA extraction of small extracellular vesicles and granulosa cells

The GCs samples and the remaining 15 μl of the resuspended pellet of FF sEVs were directed to RNA extraction. In GCs and FF sEVs 8 and 7 pools, respectively, were used per experimental group with each pool containing samples from 1-4 ovarian follicles from different cows. Total RNA was extracted according to the Trizol reagent (Thermo Fisher Scientific) manufacturer's instruction and with the addition of 1.33 μL the coprecipitate GlycoBlue (Thermo Fisher Scientific) to the aqueous phase before RNA precipitation, as previously described by (Silveira et al., 2018; Gonella-Diaza et al., 2021) with minimal modifications. Total RNA concentrations were analyzed using spectrometry (NanoDrop™ One/OneC Microvolume UV-Vis Spectrophotometer, Thermo Fisher Scientific). The RNA was treated with DNase I (Invitrogen; Carlsbad, CA) according to the manufacturer's instructions to avoid DNA contamination.

#### Reverse transcription and quantitative qRT-PCR analysis of mRNAs in small extracellular vesicles and granulosa cells

For cDNA synthesis, reverse transcription was performed with the commercial High-Capacity cDNA Reverse Transcription Kit (Thermo Fisher Scientific). Approximately 100 ng and 1000 ng of the total RNA from FF sEVs and GCs, respectively, was incubated with 10x RT Buffer, 25x dNTP mix (100 mM), 10x RT Random Primers, MultiScribe Reverse Transcriptase (50 U/μL) and nuclease-free water at 25 °C for 10 minutes and at 37 °C for 120 min, followed by 5 minutes at 85 °C to stop the reaction.

For quantitative RT-PCR, SYBR Green RT-PCR Master Mix (Thermo Fisher) was used according to the manufacturer’s instructions. The total volume of the reaction mixture was 10 μL, and it contained 5 μL of 2× Power SYBR Green PCR Master Mix, nuclease-free water, 13.5 ng or 30 ng of cDNA from FF sEVs or GCs per reaction analyzed, respectively, and 1.5 μL of 0.5 μM forward + reverse primers. qRT-PCR reactions were performed using QuantStudio 6 Flex (Applied Biosystems) equipment. The reactions were exposed to 95 °C for 10 min, 40 cycles of 95 °C for 15 s, and 60 °C for 60 s. This was followed by melting curve according to the manufacturer’s instructions. We considered the mRNA present when cycle threshold (CT) was less than 37 cycles with adequate melting curves. CT was normalized using *GAPDH* for FF sEVs and the geometric mean of *YWHAZ* and *GAPDH* for GCs, obtained in duplicate, with the method described by (Silveira et al., 2017). The ΔCt method was used to calculate the relative expression of the mRNAs individually (Schmittgen and Livak, 2008). Next, data were transformed by 2^-ΔCt^ method. The list of primers is indicated in Table 1.

#### Statistical analysis

The mRNA expression data such as FF sEVs concentration and size were analyzed using the one-way ANOVA and the averages were compared using the post-hoc Tukey’s test, with a significance level of 5% following normality criteria. The statistical analyses were performed using Prism-4 software (version 4.03, Graph Pad, San Diego, CA).

### Experiment 2

#### Collection of granulosa cells and follicular fluid

Ovarian samples were obtained from a local slaughterhouse and transported in warmed (37 °C) physiological saline solution (0.9% NaCl). Upon arrival, the ovaries were washed with a physiological saline solution and subjected to a 70% ethanol rinse, followed by three washes in warmed saline solution. Follicular aspiration was performed using a vacuum pump (GenX International; Guelph, ON, Canada) set to approximately 50 mmHg, with sterile 50 mL conical tubes (CELLTREAT® Scientific Products; Pepperell, MA, United States) attached to 18-gauge needles (Covidien Monoject™; Mansfield, MA, United States). Ovarian follicles were grouped by diameter size: 3-6 mm (Small) and 8-14 mm (Large). The follicular aspirate was left at a warm temperature for approximately 10 minutes, allowing the cumulus-oocyte complexes to precipitate at the bottom of the tubes. These were collected in new 15 mL tubes (Thermo Fisher Scientific; Waltham, MA, United States) containing warmed PBS−. The FF containing GCs was then homogenized via repetitive pipetting and centrifuged at 500 × g for 7 minutes to collect GC pellets, with the supernatants separated to isolate the FF sEVs.

The GCs pellets were resuspended in 350 µL of red blood cell (RBC) lysis buffer (Sigma-Aldrich; St. Louis, MO, United States) for 3 minutes. To stop the RBC lysis, the cells were washed twice with Dulbecco’s Modified Eagle’s Medium/Ham’s Nutrient Mixture F12 (DMEM F12) (Sigma-Aldrich; St. Louis, MO, United States), supplemented with 10% exosome-depleted fetal bovine serum (FBS) (System Biosciences; Palo Alto, CA, United States), 1% Penicillin-Streptomycin (Sigma-Aldrich; St. Louis, MO, United States), and 1% Amphotericin B solution (Sigma-Aldrich; St. Louis, MO, United States). The samples were centrifuged at 500 × g for 5 minutes, the supernatant discarded, and the cell pellets were washed with PBS− before being resuspended in DMEM F12 supplemented medium.

The resuspended cells were counted, and viability was determined using a hemocytometer (Hausser Scientific; Horsham, PA, United States) with the trypan blue exclusion method (Sigma-Aldrich; St. Louis, MO, United States). Approximately 4 pools of samples were created, each containing samples from about 20 ovaries, to achieve the required volumes for analysis, whether for GC or sEVs samples.

#### Granulosa cells culture and harvest

To better understand whether GCs are an additional source of FF sEVs containing decreasing levels of LHR mRNA, cell cultures were established and the sEVs secreted into the culture medium (ME-sEVs) were analyzed. GCs were seeded in 96-well culture plates (United States Scientific, Inc.; Ocala, FL) at a density of 10,000 cells per well, each well containing 100 µL of culture medium. The cultures were maintained at 37 ºC in an atmosphere of 95% humidity and 5% CO2. After 24 hours, the cells were washed with 100 µL of PBS− to remove dead cells and cell clusters. After 48 hours, the cells were washed again with PBS− and detached by adding 100 µL of 0.25% EDTA-Trypsin (Sigma-Aldrich). The cells were incubated for 3 minutes, and detachment was confirmed under a microscope. To stop the trypsin reaction, 100 µL of DMEM F-12 with serum was added, and the plates were incubated for an additional 4 minutes. The cells were then collected and centrifuged at 750 × g for 5 minutes. The resulting pellet was washed with PBS−, and the cells were stored at -80 ºC.

#### Isolation of small extracellular vesicles from follicular fluid and cell culture medium

In order to isolate the FF sEVs and the ME-sEVs, the samples were submitted to serial centrifugations. Firstly, the samples were centrifuged at 500 x g for 10 minutes at 4ºC to remove the cells. The pellet was discarded, and the supernatant was submitted to a new centrifugation of 3,000 x g for 10 minutes at 4ºC in order to remove the cellular debris. Finally, the supernatant was centrifuged at 17,200 x g for 30 minutes at 4ºC, removing large microvesicles. The samples then were filtered through a 0.22 μm sterile syringe filter (Sigma-Aldrich; St. Louis, MO, United States), to remove particles greater than 220 nm in diameter. The next steps were the ultracentrifugation, which were performed twice at 120,000 x g for 70 minutes using the Optima XE-90 Ultracentrifuge (Beckman Coulter; Pasadena, CA, United States), in 5.2 mL UltraClear™ Centrifuge Tubes (Beckman Coulter; Pasadena, CA, United States) and the Beckman SW 55Ti rotor (Beckman Coulter; Pasadena, CA, United States). At the final, the FF sEVs and ME-sEVs were resuspended in 500 μL of PBS− and stored in -80º C until further analysis.

#### Nanoparticle tracking analysis

The size and concentration of the FF sEVs and ME-sEVs samples were characterized using nanoparticle tracking analysis (NTA). Briefly, 5 µL of purified sEVs were diluted in 995 µL of PBS− and loaded into the ZetaView® QUATT 4 Nanosight Instrument (Particle Metrix; Ammersee, Germany), which is equipped with four distinct lasers (405/488/520/640 nm sources). Video measurements were recorded for each sample, and 11 positions were analyzed using the Zetaview software (version 8.05.12 SP1).

#### Total RNA extraction of small extracellular vesicles and granulosa cells

The total RNA was isolated from 4-5 pools (from at least 20 ovaries each) of GCs and FF sEVs and ME-sEVs using the miRNeasy® mini kit (Qiagen; Hilden, Germany) and Exosomal RNA Isolation Kit (Norgen Biotek Corp), respectively. For all the samples, the RNase-free DNase (Qiagen; Hilden, Germany) was used for DNA digestion. The RNA extraction kits were used according to the manufacturers’ instructions. In order to measure the RNA concentration and integrity, we used the NanoDrop 2000 Spectrophotometer (Thermo Scientific; Waltham, MA, United States). Finally, the isolated RNA samples were stored in -80 ºC.

#### Reverse transcription and quantitative qRT-PCR analysis of the mRNAs in small extracellular vesicles and granulosa cells

The relative quantification of *LHR* was measured by qRT-PCR in GCs, ME-sEVs and FF sEVs with at least 4 replicates. The housekeeping genes used to the geometric mean to normalize the results were the *ß-ACT* and the *GAPDH* for GCs, and only *GAPDH* for EVs. In order to perform the qRT-PCR, the cDNA was synthesized by reverse transcribing from the same amount of RNA in each sample. For this, the First-Strand cDNA synthesis kit (Thermo Fisher Scientific), according to the manufacturers’ instructions. The qRT-PCR reaction was performed with 20 µl in total volume, in which we used 0.5 µl of the Forward and Reverse sequence of the primer (10 µM), totalling 1 µl. A volume of 10 µl of the 1xSYBR Green Master Mix (Bio-Rad) was also used to perform the reaction, with 2 µl of the cDNA and 7 µl of nuclease free water. The negative controls had a total of 9 µl of nuclease free water in order to substitute the 2 µl of cDNA. The total amount of cDNA used for each reaction of qRT-PCR for sEVs and GCs was 10 ng and 30 ng, respectively. The thermocycling conditions were applied as: initial denaturation at 95 °C for 3 minutes, followed by 40 cycles of amplification at 95 °C for 15 seconds, and 60 °C for 60 seconds. The qRT-PCR was performed using the CFX96 Touch Real Time PCR Detection System (Bio-Rad; Hercules, CA, United States) and data was analyzed using the comparative Ct method (Schmittgen and Livak, 2008). The melting curve was generated and analyzed to check the amplification specificity. The gene-specific primers were designed utilizing the Primer-Blast (https://www.ncbi.nlm.nih.gov/tools/primer-blast/) and the list of primers is indicated in Table 1.

#### Statistical analysis

In Experiment 2, for the comparison of sEVs concentration and size, and *LHR* levels in GCs, FF sEVs and ME-sEVs, a Two Tailed student's t-test was performed, also with a significance level of 5%. The statistical analyses were performed using Prism-4 software (version 4.03, Graph Pad, San Diego, CA).

## Results

### Characterization of sEVs from Small, Medium, and Large Follicles from Stage 2 and 3 Ovaries Reveals Similar Concentration and Size

**Figure 2:**
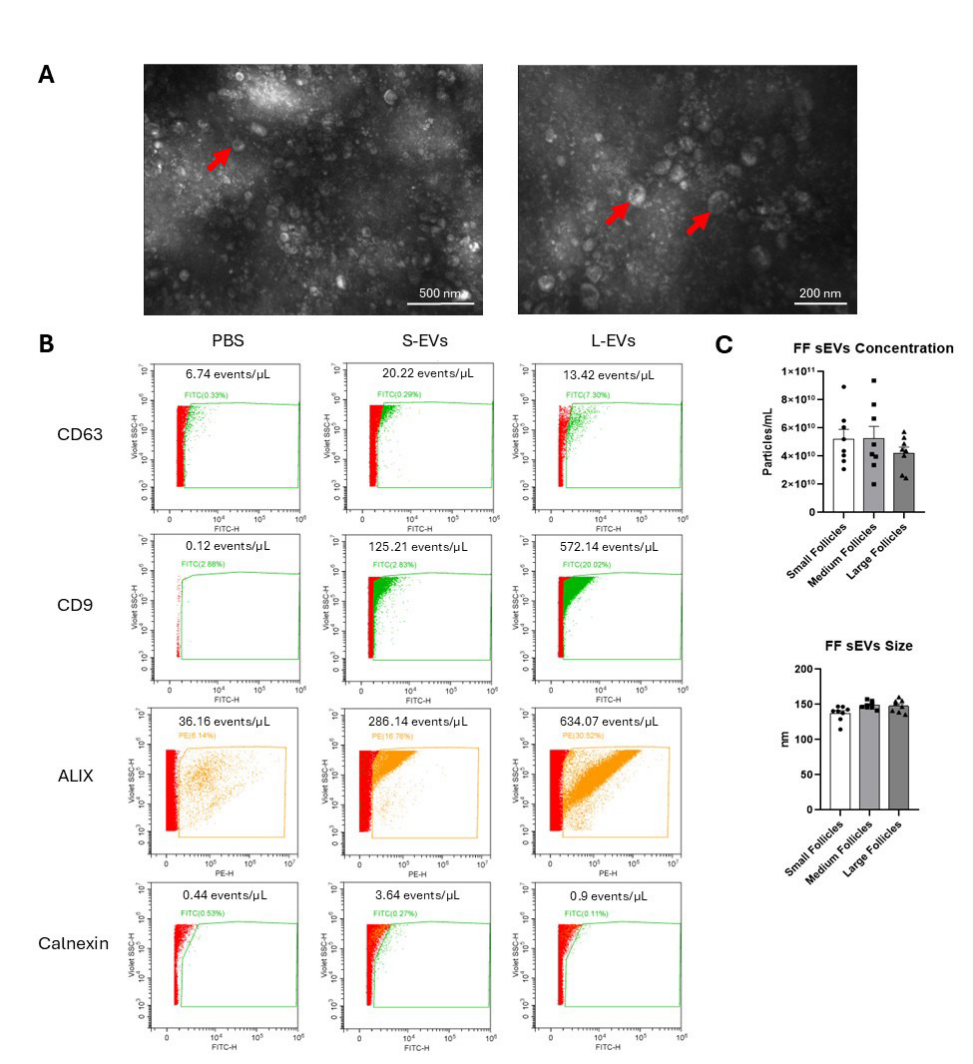
Characterization of sEVs isolated from follicular fluid. From Experiment 1: (A) Transmission electron microscopy showing sEVs around 50–150 nm in diameter and their cup-shape appearance (indicated by the red arrow). The magnification of the electron microscopy images was 100,000x. (B) Nano-flow cytometry analysis confirmed the presence of sEVs markers, including CD63, CD9, and ALIX, while detecting minimal calnexin signal, a known cellular marker. (C) Nanoparticle tracking analysis showing no difference in particles concentration and particles size among the groups (P > 0.05): small, medium and large follicles. Solid bars represent the group mean, error bars represent the SEM, and each dot represents one sample corresponding to the biological replicate.

In Experiment 1, in order to confirm the presence of the sEVs by the method used to isolate them, three different techniques were performed. The first method was Transmission Electron Microscopy (TEM), which showed in images the presence of the sEVs in follicles samples (Figure 2A). The nano-flow cytometry was the second method utilized in order to identify specific proteins not found in cells but found in sEVs, such as CD9, CD63 and ALIX, showed in the image. Additionally, Calnexin detection in sEVs was minimal, with signal levels comparable to those observed in the PBS control (Figure 2B). Finally, the NTA was performed in order to measure the concentration and size of the sEVs samples. This way, it was demonstrated that we had sEVs in both samples (which size is between 30 and 150 nm). The concentration and size of the sEVs from small, medium and large follicles did not show any statistical difference (Figure 2C).

### From ovaries at random stages of the estrous cycle, small and large follicles exhibited similar sEV concentrations

**Figure 3:**
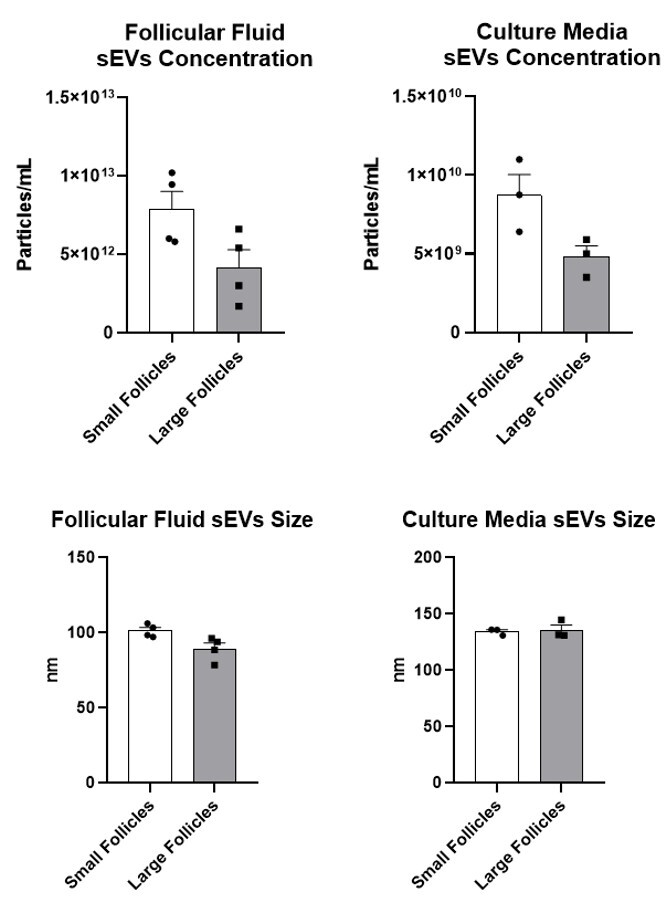
Nanoparticle tracking analysis showing no statistical difference in both FF (P = 0.060) and media sEVs (P = 0.059) concentrations and sizes in Experiment 2. Solid bars represent the group mean, error bars represent the SEM, and each dot represents one sample corresponding to the biological replicate.

In Experiment 2, in order to verify the efficacy of the isolation of the sEVs from small and large follicles from ovaries of any stages of the estrous cycle, the NTA was performed. The concentration of the sEVs from small and large follicles showed a tendency of statistical difference in both FF (P = 0.060) and media sEVs (P = 0.059) samples. In addition, we did not observe any difference in media sEVs size (Figure 3).

### *LHR* is differentially regulated based on follicle size in granulosa cells and extracellular vesicles from random ovaries or from stages 2 and 3 of the estrous cycle

**Figure 4:**
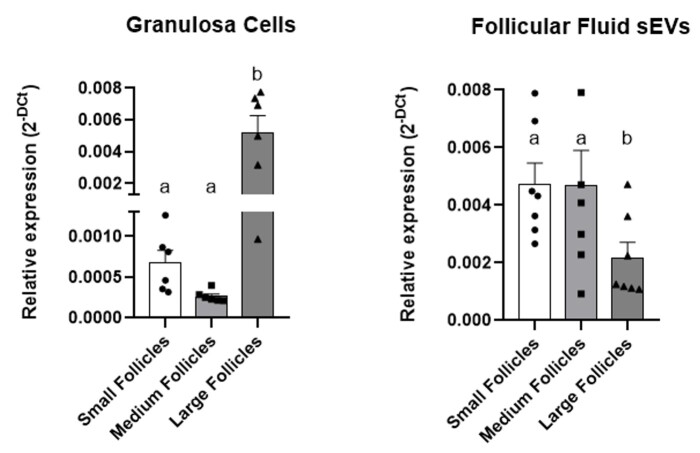
*LHR* relative expression in GCs and FF sEVs respectively from small, medium and large follicles from Experiment 1. Different letters indicate statistical difference, considered when P ≤ 0.05. Solid bars represent the group mean, error bars represent the SEM, and each dot represents one sample corresponding to the biological replicate.

**Figure 5:**
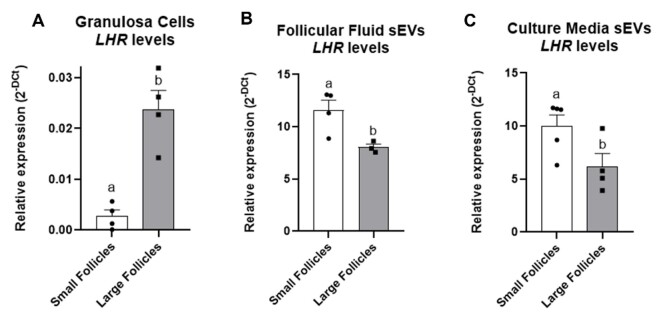
From Experiment 2, *LHR* relative expression in: (A) GCs from small and large follicles; (B) sEVs from small and large follicles; and (C) sEVs from small and large follicles GCs in the culture media. Solid bars represent the group mean, error bars represent the SEM, and each dot represents one sample corresponding to the biological replicate.

To investigate a possible relation in the *LHR* levels between the GCs and the FF sEVs, its transcripts were measured by qRT-PCR. In Experiment 1, it was demonstrated that the large follicles GCs had higher levels of the *LHR* when compared to both small (P = 0.0018) and medium (P = 0.027) follicles GCs (Figure 4). In Experiment 2, a similar result was observed being the *LHR* levels also increased in large follicles compared to GCs from small follicles GCs (P = 0.001) (Figure 5A).

On the other hand, we observed the opposite correlation in the sEVs. In experiment 1, higher levels of the *LHR* were found in sEVS from small (P = 0.022) and medium (P = 0.015) follicles when compared to large ones (Figure 4). Similarly, in Experiment 2, the sEVs from small follicles showed a higher expression (P = 0.024) of the *LHR* in comparison to large follicles sEVs (Figure 5B).

### Granulosa cells cultured *in vitro* secrete EVs following the same pattern as *in vivo*

In Experiment 2, to better understand whether the GCs themselves could secrete FF sEVs containing LHR mRNA, GCs from small and large follicles were cultured. sEVs secreted by GCs from small follicles exhibited higher LHR mRNA expression (P = 0.050) compared to those secreted by GCs from large follicles (Figure 5.C), following the same pattern observed *in vivo*.

## Discussion

In the present study, we identified *LHR* mRNA in GCs and, for the first time, in FF sEVs of ovarian follicles ranging from 3 to 14 mm in diameter. Experiment 1, which primarily used *Bos taurus indicus* samples, revealed higher levels of *LHR* mRNA in sEVs from small and medium follicles compared to large follicles. Experiment 2, involving *Bos taurus taurus* samples, confirmed the same pattern. The consistency across both subspecies suggests that this correlation is a standard feature of bovine physiology. Additionally, GCs in culture were shown to secrete sEVs with cargo patterns consistent with *in vivo* observations, with higher *LHR* expression in sEVs from the culture media of GCs from small follicles. This finding provides strong evidence that these sEVs are secreted by GCs and may facilitate the transfer and acquisition of *LHR* by other GCs during follicular development.

The absence of LH receptors has been associated with genetic issues leading to fertility problems in both cattle (Arnhold et al., 1999) and humans (Ferguson, 2017). Given the crucial role of LH, substantial research has focused on factors influencing *LHR* expression and roles during follicular development in GCs. Various molecules have been identified as regulators of the *LHR* gene, either repressing (Santos et al., 2022; Nair et al., 2002; Ereno et al., 2015) or promoting its overexpression (Li et al., 2024). Nonetheless, the precise mechanisms by which *LHR* levels are increased in GCs of dominant follicles remain unclear.

Molecules such as LRBP and bta-miR-222 have been described as important regulators of *LHR* expression. LRBP is an mRNA-binding protein that interacts with the coding region of *LHR* mRNA, repressing its translation, particularly around the time of follicular deviation (Nair et al., 2002; Ereno et al., 2015). Similarly, bta-miR-222 modulates *LHR* expression, mainly in preovulatory follicles (Santos et al., 2018). Interestingly, the levels of both LRBP (Ereno et al., 2015) and bta-miR-222 (Salilew-Wondim et al., 2014) decrease in GCs from dominant follicles. This decrease suggests that dominant follicles actively suppress inhibitory factors to allow the accumulation of LHR.

In light of our findings, the increased presence of *LHR* mRNA in FF-derived sEVs—especially from smaller antral follicles—raises the hypothesis that these vesicles may act early in the process, temporally compensating for the presence of inhibitory regulators before their natural downregulation occurs. Furthermore, it is possible that once a threshold level of *LHR* mRNA—or its translated protein—is reached within granulosa cells, this accumulation or the LH receptor activation at higher levels may contribute to the downregulation of repressive factors such as LRBP and bta-miR-222. This would suggest a self-reinforcing mechanism in which the early delivery of *LHR* mRNA via sEVs not only enhances LH responsiveness but also promotes an internal shift toward follicular dominance by repressing negative regulators.

**Figure 6:**
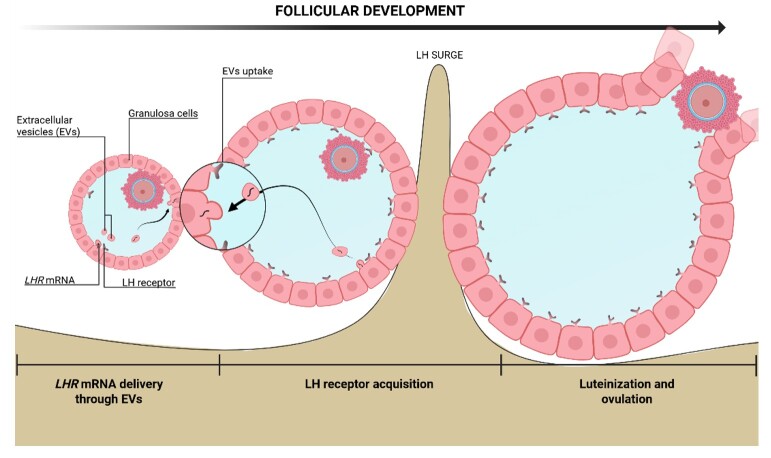
Hypothetical model of the *LHR* mRNA acquisition mechanism in GCs during follicular development. The mRNA is encapsulated by FF sEVs secreted by GCs that already contain the receptor. These FF sEVs are then delivered to GCs that do not contain the receptor, enabling these cells to acquire the receptor through sEV-mediated delivery. This way, the GCs are able to have homogenous levels of the receptor, enabling the GCs to luteinize and the follicle to ovulate.

These observations support a broader model in which the accumulation of *LHR* mRNA, partially mediated by sEVs, may trigger a coordinated shift in the follicular environment. Building upon this hypothesis, we propose a mechanism wherein some granulosa cells initially acquire endogenous *LHR* mRNA. Upon reaching a threshold in LHR expression, these cells begin to secrete sEVs containing *LHR* mRNA. This allows other GCs within the follicle to attain higher and more uniform levels of *LHR* mRNA, even if they are not in proximity to the secreting cells. Consequently, the follicle becomes primed for dominant, enhancing its responsiveness to the LH surge and leading to ovulation (Figure 6).

Further investigations are needed to confirm this hypothesis, including testing whether supplementation with sEVs from small follicles increases *LHR* mRNA and protein levels in granulosa cells, as well as assessing the expression of known inhibitory regulators such as LRBP and bta-miR-222. Additionally, this mechanism could be further explored by blocking or activating LH signaling pathways and evaluating the downstream effects, such as granulosa cell luteinization and the secretion of luteinization-associated hormones. These insights highlight the potential application of sEVs in promoting follicular growth and development, with possible implications for improving assisted reproductive technologies and treating genetic disorders in both humans and cattle.

## Conclusions

In conclusion, this study demonstrates for the first time that FF sEVs from both *Bos taurus taurus* and *Bos taurus indicus* contain *LHR* mRNA. Additionally, the inverse compensatory mechanism observed for both the bovine subspecies in *LHR* transcripts within FF sEVs and GCs from follicles of varying sizes strongly supports the hypothesis that these FF sEVs play a critical role in facilitating LH receptor acquisition in GCs as follicle grow towards dominance. Nonetheless, further studies are required to confirm that this pattern is directly attributable to the delivery function of sEVs, and to determine whether this ultimately contributes to LH receptor development in GCs.

## Data Availability

Research data is available in the body of the article.
